# The Dark Kinase Knowledgebase: an online compendium of knowledge and experimental results of understudied kinases

**DOI:** 10.1093/nar/gkaa853

**Published:** 2020-10-20

**Authors:** Matthew E Berginski, Nienke Moret, Changchang Liu, Dennis Goldfarb, Peter K Sorger, Shawn M Gomez

**Affiliations:** Department of Pharmacology, University of North Carolina at Chapel Hill, Chapel Hill, NC 27599, USA; Laboratory of Systems Pharmacology, Department of Systems Biology, Harvard Program in Therapeutic Science, Harvard Medical School, Boston, MA 02115, USA; Laboratory of Systems Pharmacology, Department of Systems Biology, Harvard Program in Therapeutic Science, Harvard Medical School, Boston, MA 02115, USA; Department of Cell Biology and Physiology, Washington University in St. Louis, St. Louis, MO 63110, USA; Institute for Informatics, Washington University in St. Louis, St. Louis, MO 63110, USA; Laboratory of Systems Pharmacology, Department of Systems Biology, Harvard Program in Therapeutic Science, Harvard Medical School, Boston, MA 02115, USA; Department of Pharmacology, University of North Carolina at Chapel Hill, Chapel Hill, NC 27599, USA; Joint Department of Biomedical Engineering at the University of North Carolina at Chapel Hill and North Carolina State University, Chapel Hill, NC 27599, USA

## Abstract

Kinases form the backbone of numerous cell signaling pathways, with their dysfunction similarly implicated in multiple pathologies. Further facilitated by their druggability, kinases are a major focus of therapeutic development efforts in diseases such as cancer, infectious disease and autoimmune disorders. While their importance is clear, the role or biological function of nearly one-third of kinases is largely unknown. Here, we describe a data resource, the Dark Kinase Knowledgebase (DKK; https://darkkinome.org), that is specifically focused on providing data and reagents for these understudied kinases to the broader research community. Supported through NIH’s Illuminating the Druggable Genome (IDG) Program, the DKK is focused on data and knowledge generation for 162 poorly studied or ‘dark’ kinases. Types of data provided through the DKK include parallel reaction monitoring (PRM) peptides for quantitative proteomics, protein interactions, NanoBRET reagents, and kinase-specific compounds. Higher-level data is similarly being generated and consolidated such as tissue gene expression profiles and, longer-term, functional relationships derived through perturbation studies. Associated web tools that help investigators interrogate both internal and external data are also provided through the site. As an evolving resource, the DKK seeks to continually support and enhance knowledge on these potentially high-impact druggable targets.

## INTRODUCTION

Protein kinases take a central position in many aspects of cellular signaling, with established roles in cellular growth, differentiation, migration and apoptosis. With well over 500 members (1,2), kinases also represent one of the largest gene families within humans (3,4). Likely arising from their central role in numerous functional processes, kinase dysfunction is similarly linked to pathologies including cancer, infectious disease and immune disorders. Protein kinases also have the beneficial property of being highly druggable by both allosteric and competitive inhibitors. This druggability, combined with their direct or indirect role in numerous diseases, establishes kinases as a key target for current and future therapeutic development efforts.

Despite their recognized importance, we lack an understanding of the functional role of roughly one-third of kinases (5). This gap in knowledge is presumably due to highly related and potentially redundant gene function of kinase family members, redundancy in the signaling pathways they are part of, and tissue or developmental stage specificity. However, recent work is now demonstrating their potential importance in multiple disease contexts (1,6–8). The potential impact of better understanding these druggable and disease-relevant targets has thus led to the establishment of NIH’s Illuminating the Druggble Genome (IDG) Program (9). This program seeks to establish a better understanding of the function of poorly studied genes in three major, druggable families, including GPCRs, ion channels and kinases. Specifically centered on kinases, the Kinase DRGC (Data and Resource Generating Center) component of the IDG program is focused on generating, systematizing and disseminating knowledge about dark kinases, the biological networks in which they function, and their connections to cellular phenotypes and human disease.

As part of the Kinase DRGC effort, the Dark Kinase Knowledgebase (DKK) is being developed to collect and disseminate DRGC data covering the 162 kinases that have been identified by NIH as being poorly understood or are otherwise ‘dark kinases.’ The data being disseminated through the DKK arise from efforts in the Kinase DRGC focused on generating new data, both experimental and computationally derived, that provides functional context for each of these kinases. Combined with relevant external data sets and cataloguing of existing resources, the Kinase DRGC is attempting to integrate and synthesize these primary data into functional biochemical and biological information that will help researchers pursue more sophisticated investigations. This support for external researchers is, in part, being implemented through the development of genetic and chemical tools, which are being made available through the DKK.

Here, we describe the Dark Kinase Knowledgebase, the initial sets of data that are being collected and presented, as well as associated web tools that help to place these kinases in a functional context. As the IDG Program advances and DRGC efforts evolve, we expect the data and tools presented in the DKK to grow into a valuable resource that supports research into these novel druggable targets with significance in human health and disease.

## MATERIALS AND METHODS

The Dark Kinase Knowledgebase uses a collection of open source libraries and tools to both organize and display the data sets associated with the resource.

### Web and data analysis tools

The DKK server runs Red Hat Linux, with the Apache HTTP server providing access to the web resources. On top of Apache, incoming requests to the darkkinome.org website are handled by the Dancer2 web application framework. The search functionality uses the Text::Fuzzy Perl module to allow for partial matching of kinase names when searching the website. The content of each page served on darkkinome.org is stored using the Template Toolkit system and customized depending on the types of data available for that kinase. The D3 javascript library was used to make the bubble chart displayed on the home page. The HTTPS certificate is provided by the ‘Let’s Encrypt’ service.

All of the custom figures displayed on the individual kinase pages were produced using R and the ggplot plotting library (10). The dark kinase expression application was built using the R Shiny framework and served using Shiny Server provided by R Studio. The anatogram images showing the highlighted expression locations are built using gganatogram (11). The source code for both the dark kinase knowledgebase and the expression application are available through github (see Code and Data Availability section).

### DRGC data sets

The IDG-Kinase consortium is collecting several data sets that interrogate the functions of understudied kinases. One area where new tools are being identified by the consortium, involves small molecule inhibitors of kinase activity and corresponding information about the small molecule’s activity across the entire kinome. To enable mass spectrometry-based efforts to quantify the concentration of dark kinases in lysate samples, the consortium is determining parallel reaction monitoring (PRM) peptides that match with each dark kinase. The PRM peptide data set includes the peptide associated with the kinase along with information about the peptide’s performance in a standard curve assay and the limit of detection for the peptide. Finally, we have also made available a set of physical interaction networks centered around several of the dark kinases collected through affinity purification mass spectrometry. Each of these data sets are expanding as the groups that make up the IDG-Kinase consortium continue their efforts to test new tools and make new data available.

All of the data sets presented in the DKK were collected using protocols appropriate for the specific data type. The PRM peptides were selected from unique peptides present in each kinase and then assayed using the guidelines developed through the CPTAC consortium (12). The small molecule inhibitor identification efforts were guided by criteria developed in the inhibitor community (13). These include broad based kinome scanning through the KinomeScan assay available through DiscoverX with promising compounds being assayed with NanoBRET probes. We have also put detailed methods concerning NanoBRET probe usage and associated tracer compounds on the DKK (https://darkkinome.org/NanoBRET). The physical interaction networks were gathered using affinity purification mass spectroscopy as described previously (14).

### Outside data sets

Data sets from the GTex consortium (15) and the Human Proteome Map (16) were downloaded from their respective data portals. We used the median gene-level TPM by tissue counts provided by the GTex consortium and the relative abundance counts provided by the Human Proteome Map. We extracted the kinases from each of these data sets and then determined, on a per tissue basis, the relative expression of each dark kinase. For visualization, each of the tissue types listed in the GTex consortium and Human Proteome map were aligned to the categories provided by the gganatogram package. Data concerning cancer mutation, mRNA levels and copy number variations were downloaded from the TCGA firebrowse website using the FirebrowseR package (17). We also display molecular structures from PDB (18,19) that include a dark kinase. These structures are displayed using the NGL viewer (20,21).

## RESULTS

The web presence for the Kinase DRGC can be divided into two parts: the primary website at darkkinome.org and a collection of standalone tools. This paper highlights the collection of resources made available through darkkinome.org along with a linked dark kinase expression browser we have developed.

### Overall website layout

The knowledgebase is divided into several sections to simplify finding and displaying the data collected through the Kinase DRGC. These include a page detailing the data available through the website along with links for downloading the data. We have also collected and provided short descriptions of the tools that have been built by the Kinase DRGC or have a direct connection to the website. Finally, we also have a listing of people and publications associated with the project. Each kinase has a page dedicated to information about that kinase (see Sample Kinase Page Components section) and we have also added a search function to allow users to find a gene of interest.

These broad sections are complimented by specific pages dedicated to listing the inhibitor tool compounds and PRM peptides that the consortium has identified. The inhibitor tool compounds are listed in a single table to allow quick searching for a kinase of interest (https://darkkinome.org/compounds). The PRM peptides and the corresponding standard curves are also listed in a single table (https://darkkinome.org/PRM_params). Both of these data sets are cross-referenced and displayed on the appropriate individual kinase page.

### Sample kinase page components

**Figure 1.**
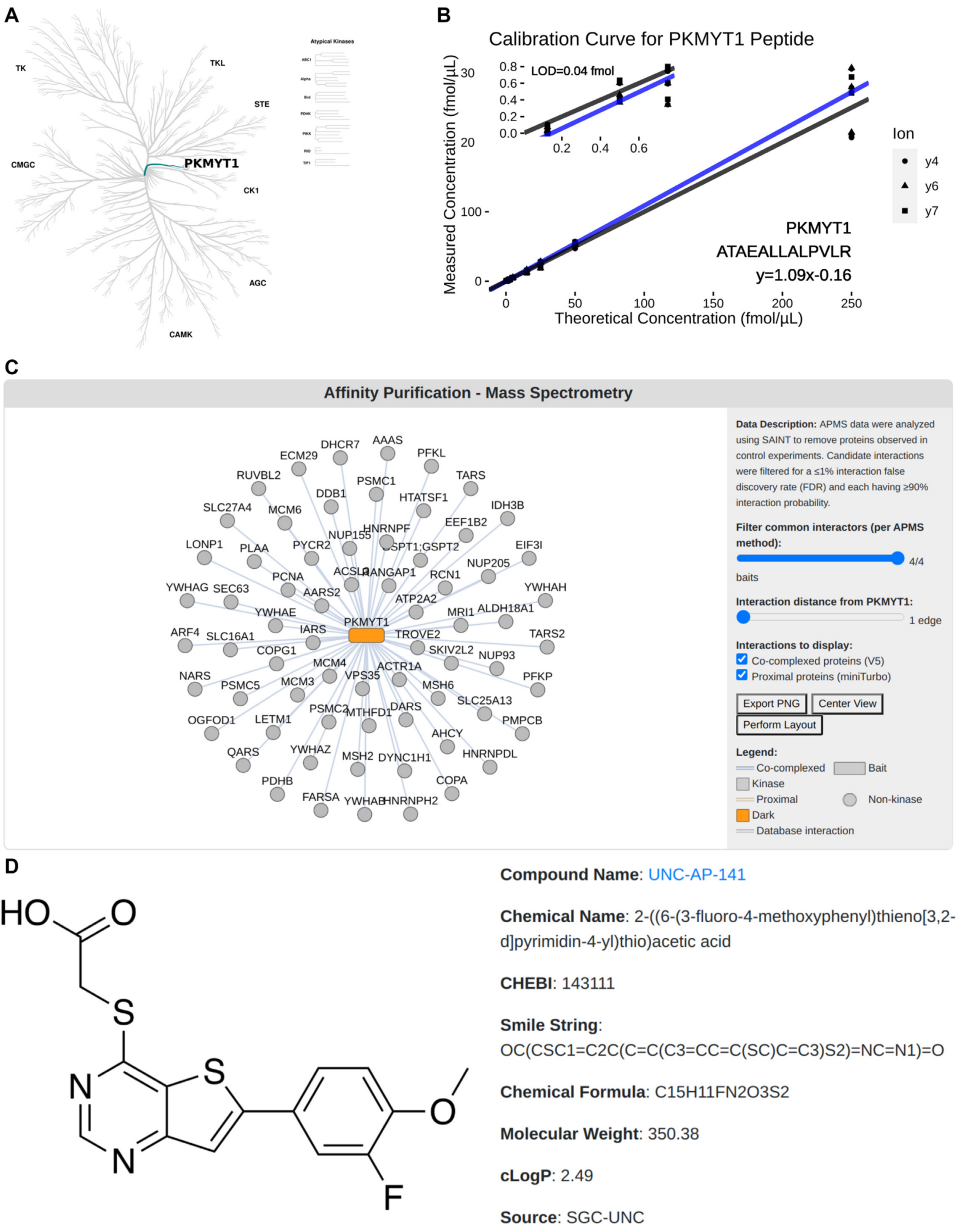
Several types of data related to dark kinases are displayed on the individual kinase pages. (**A**) Kinase tree showing the location of PKMYT1 in the canonical kinase phylogenetic tree (3) produced using CORAL (22). (**B**) Calibration curve related to a PRM peptide related to PKMYT1. (**C**) Interactive visualization of APMS physical interaction network building using Cytoscape.js (23) for PKMYT1. (**D**) Display of the tool compound for kinase STK17B.

The core of the knowledgebase is centered on providing a single page summary for each kinase of the data and related analyses provided by the DRGC, along with a limited set of relevant external data (e.g. the tissue expression distribution of the kinase as derived from GTex). The data presented on a page varies based on the types of data the Kinase DRGC has currently generated on that kinase and will be continually updated as data is collected. Examples of the types of data are shown in Figure 1 and described below.

For reference, each kinase page provides an image from CORAL (22) that shows the kinase’s position in the broader kinase family phylogenetic tree (Figure 1A). The 162 kinases under the purview of the DRGC span essentially all branches of the kinome tree, including several atypical kinases (1,24). A significant component of DRGC efforts involve development of mass spectrometry resources to support quantitative proteomics. Towards these goals, members of the DRGC are developing a set of parallel reaction monitoring peptides for each kinase. These peptides, each set of which are unique to a given kinase, enable quantification of the kinase at femtomolar resolution within a given sample. As unique peptide sequences are discovered and validated, they are presented on the associated kinase page along with the set calibration curves for use with mass spectrometry (Figure 1B).

In addition to the phylogenetic tree and PRM peptides, protein interaction networks for each kinase are also being generated, with an example being shown for PKMYT1 in Figure 1C. These networks are derived from affinity purification mass spectrometry experiments on V5-tagged kinases and proximity labeling experiments using miniTurbo-tagged kinases (14). These networks will eventually be populated for all dark kinases. The interactive visualization shown in Figure 1C further allow the user to modify a few key parameters for constructing and displaying interactions, and network diagrams can also be exported from the interface. Many of the kinase pages (∼30 kinases to date) also have summaries of characterized chemical tool inhibitor compounds (Figure 1D). The chemical tool summary section displays several key properties of the identified compounds as well as links to learn more about the compound’s inhibition properties and purchase the compound.

A number of other databases have highly relevant and complementary information about a given kinase, and so for each kinase we have outgoing links to resources including Pharos (https://pharos.nih.gov/) (25), Gene Cards (https://www.genecards.org/) (26) and the Monarch Initiative (https://monarchinitiative.org/) (27). We have similarly summarized TCGA data for each kinase through heatmaps that provide a visualization on mutations, copy number variation and mRNA expression levels of the kinase across multiple cancer types. We are further providing functional interaction networks for each dark kinase, for example using data integrated through INDRA (1,28,29). A summary of the highlights from our Dark Kinase expression browser (described further below) is also displayed with corresponding links to the full application. As data specific to each understudied kinase is generated by the Kinase DRGC, kinase pages will be updated and further integrated with relevant external data sets.

### DKK expression browser

**Figure 2.**
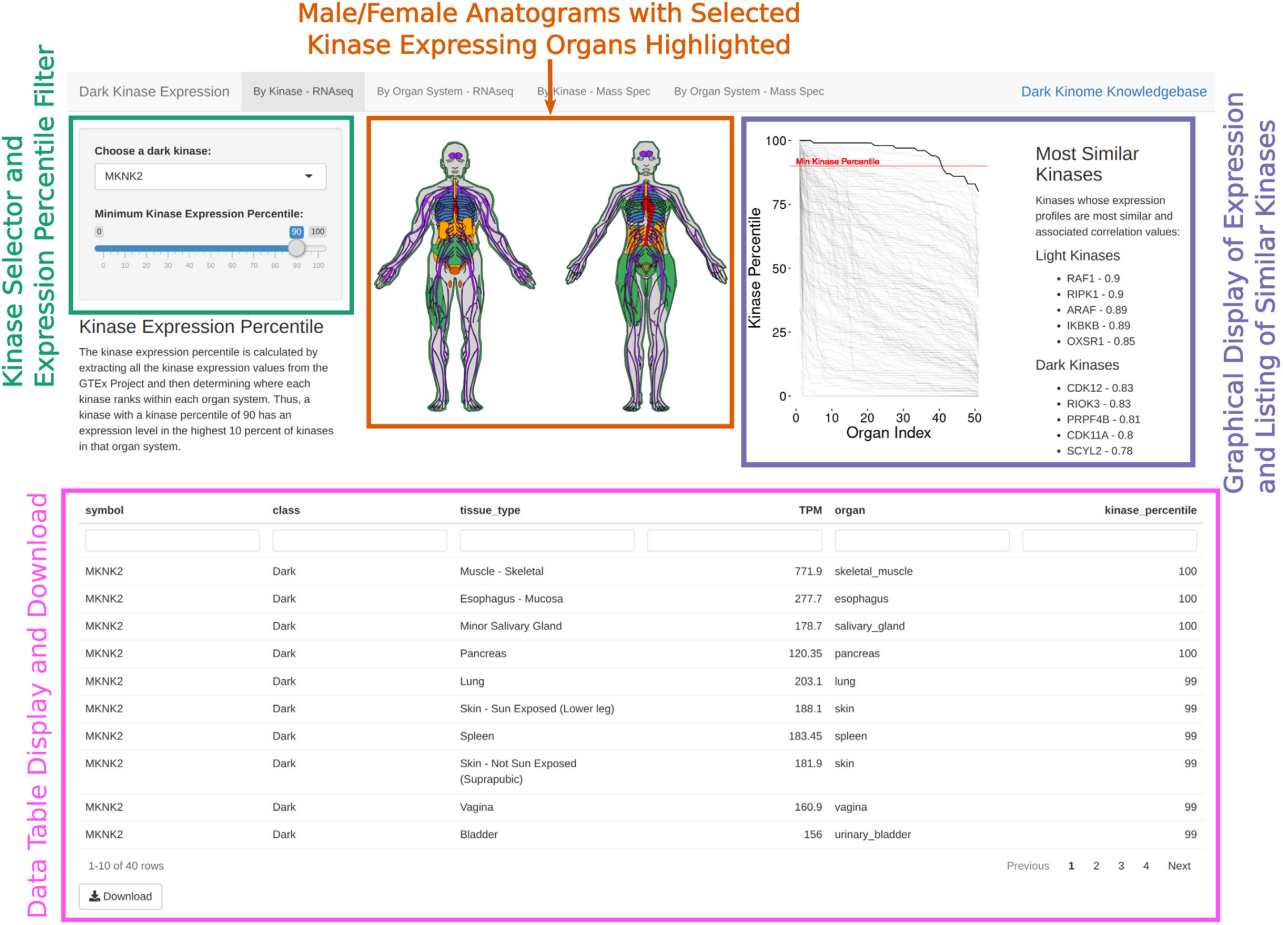
Sample screenshot from the Dark Kinase Expression browser (http://expression.darkkinome.org) with MKNK2 selected. Individual sections of the page are highlighted with colored boxes and associated labels. Along the top edge is a tabbed interface for selecting methods of browsing (either by kinase or organ system) and the type of data (GTex Consortium or Human Protein Atlas). The colors in the anatogram figures only mark the organs expressing the selected kinase.

A common question we receive involves the question of where in the human body a given dark kinase is expressed. To help answer this question we integrated results from two comprehensive surveys of human tissues from the GTex consortium (15) and the Human Proteome Map (16). Using these two data sources we collected the RNAseq summary counts from GTex and the mass spectrometry-based survey of human protein abundance. As our focus is on placing the dark kinases into context with the rest of the kinome, we have filtered these datasets to only include kinases. This focus on kinases allows the user to compare the expression profile of a given understudied kinase in relation to other kinases that are often much better characterized. In addition, we have further contextualized measurements by converting the total read counts or mass spectrometry abundance values for all kinases into a percentile on an organ-by-organ basis (Figure 2). Using these kinome-specific data sets, we have produced an interactive data browser available through the web (http://expression.darkkinome.org/).

The browser was developed using the R shiny framework (30) and allows the user to select any of the 162 dark kinases via a dropdown menu in the upper left corner. There is also a slider to set the minimum kinase percentile displayed. When the percentile value is changed, the anatograms and the data table update to reflect this new threshold. The plot next to the anatograms displays the expression percentiles of the selected kinase with a bold line, while the remaining gray lines show the kinase expression percentiles for all remaining kinases. To further enable comparisons with the rest of the kinome, we have also calculated the correlation between kinases across organs and show the five most well correlated dark and light kinases for the selected kinase. The kinase-selection dropdown box is show in rank order of the highest average organ percentile, so the kinases highest on the list are the most expressed with those at the bottom of the list being the least expressed. MKNK2 has the highest average expression by this metric, while PSKH2 has the lowest. The data contributing to these results are displayed in a data table underneath these elements with a link to download the filtered data set in CSV format. These interface elements are replicated in the other tabs along the top of the page.

In addition to the kinase-centric view displayed on the first page, we have also created organ-centric views of this same data. This view of the data is most useful for researchers interested in a specific organ system and who would like to view the dark kinases with the highest expression in that organ. None of the organ systems show dramatically different expression variation between overall dark kinase expression profiles. Within this range of organ variation though, the brain regions make up most of the organ systems with higher dark kinase expression levels. We hope this tool will allow researchers to answer questions about the presence of dark kinases and their relation to other well-studied kinases at the organ level.

### Additional web-based tools

**Figure 3.**
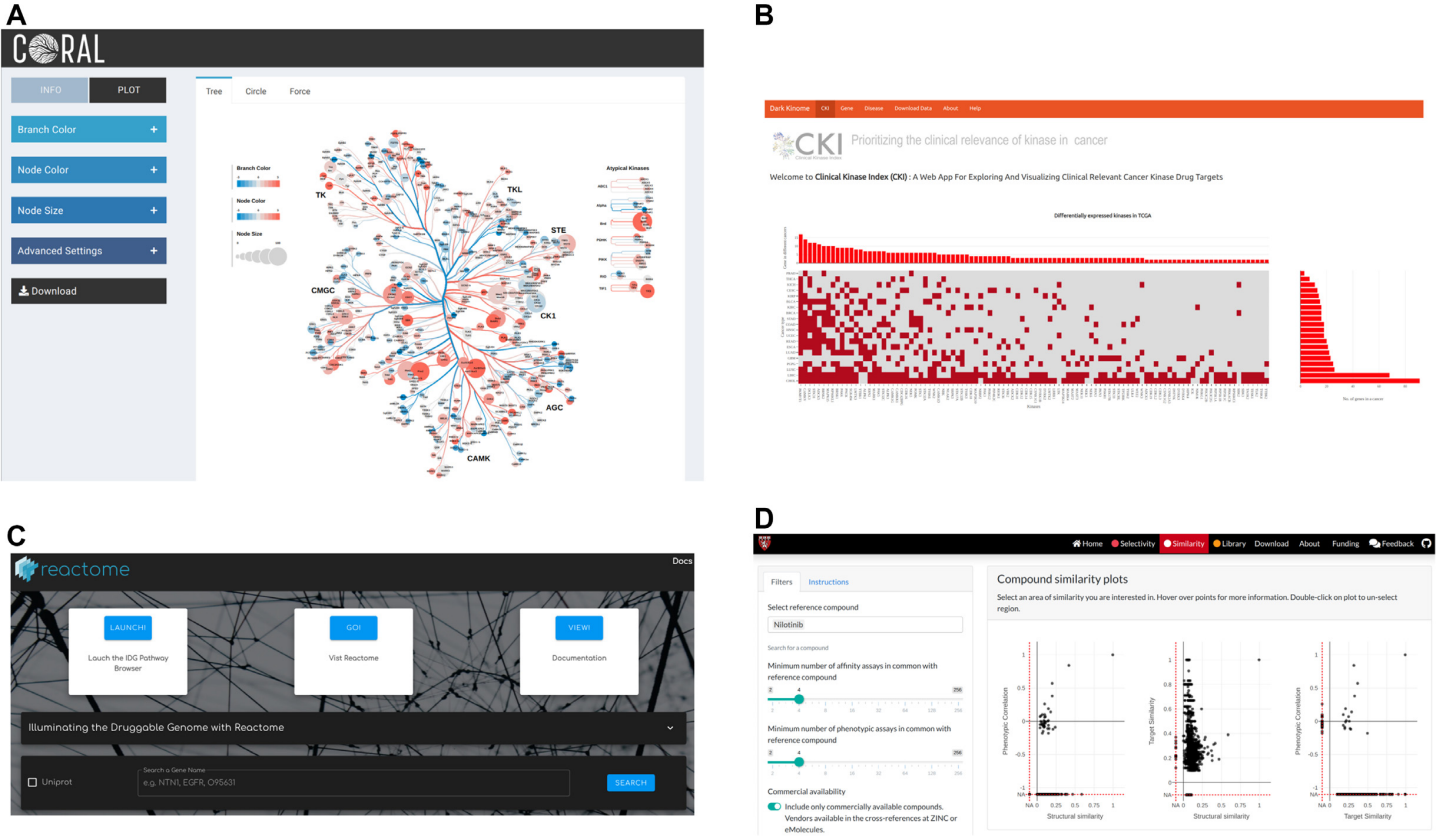
Several tools affiliated with the Dark Kinase Knowledgebase. (**A**) CORAL: the kinase tree visualization tool. (**B**) The Clinical Kinase Index, which focuses on the kinase’s role in cancer. (**C**) The IDG Reactome portal provides a window into the Reactome database centered around understudied genes. (**D**) The Small Molecule Suite toolkit for selection of kinase inhibitors

In addition to the Dark Kinome Expression Browser, several affiliated tools have also been developed by the Kinase DRGC and collaborators. These tools include:

- Visualization methods specific to the kinome (CORAL, Figure 3A) (22)
- Kinase targets in cancer (the Clinical Kinase Index, Figure 3B) (31)
- Biological pathways that include dark kinases and other understudied proteins (IDG Reactome, Figure 3C) (32)
- Collections of small molecule kinase inhibitors (Small Molecule Suite, Figure 3D) (33)

Each of these tools provide a unique set of methods and data concerning the role of understudied kinases in a range of biological contexts.

### Code and Data Availability

All of the computer code associated with the Dark Kinase Knowledgebase is available through github (https://github.com/IDG-Kinase/darkkinasekb), as is the code underlying the kinase expression browser (https://github.com/IDG-Kinase/kinase_expression). In addition to these two projects, related code produced by the consortium is listed on our github group page (https://github.com/IDG-Kinase). We also maintain a collection of data through Synapse (https://www.synapse.org/#!Synapse:syn18360482/files/) for the distribution of bulk data sets and those data types lacking standard public repositories.

## CONCLUSION AND FUTURE DIRECTIONS

The Dark Kinase Knowledgebase provides a collection of resources dedicated to improving our understanding of understudied kinases. Data underlying the DKK is derived from both experimental and computational efforts within the Kinase DRGC as well as publicly-available external data sets. A kinase-centric view provides each understudied kinase with a page dedicated to rapidly presenting information relevant to understanding potential function or to tools that can be used to experimentally investigate behavior. Similarly, associated tools such as the expression browser, place understudied kinases in the context of more well-studied kinases, further enhancing potential understanding of downstream function.

The DKK is a young and still growing resource. Ongoing Kinase DRGC efforts are focused on expanding the various data types, including chemical tools, physical and functional interaction networks, and PRM peptides, across all understudied kinases. Also, relevant external data sets will continue to be incorporated, providing an integrated picture of the current state of knowledge surrounding a particular target kinase. In addition to these data, we will further explore the development of associated web applications, similar to the expression browser, that leverage these underlying data and enable further insight into kinase function. We are also working to integrate our results with outside tools such as KinView (34). Beyond the currently represented data types, the Kinase DRGC is actively generating several types of data not currently represented on the website. For example, one such data set will detail the transcriptional changes in response to treatment with kinase inhibitors, and how these changes then modify the activities of the entire kinome. Another set of DRGC work focuses on developing and applying a novel collection of machine learning algorithms to investigate and potentially predict new small molecules capable of inhibiting kinases.

By integrating both DRGC generated data along with external data sets, the DKK is designed to help provide researchers with a concise view of the state of knowledge surrounding a given kinase. Similarly, the dissemination of DRGC relevant tools such as PRM peptides and associated calibration curves to the broader research community is a crucial goal of both the DKK and the broader IDG Consortium. As more data is generated and synthesized, we hope the DKK can provide greater insight into the function of these currently neglected, druggable molecular targets.
